# Selenium Supplementation in Fish: A Combined Chemical and Biomolecular Study to Understand Sel-Plex Assimilation and Impact on Selenoproteome Expression in Rainbow Trout (*Oncorhynchus mykiss*)

**DOI:** 10.1371/journal.pone.0127041

**Published:** 2015-05-15

**Authors:** Davide Pacitti, Muhammad M. Lawan, John Sweetman, Samuel A. M. Martin, Jörg Feldmann, Christopher J. Secombes

**Affiliations:** 1 Scottish Fish Immunology Research Centre, Institute of Biological and Environmental Sciences, University of Aberdeen, Aberdeen, United Kingdom; 2 Trace Element Speciation Laboratory, Department of Chemistry, University of Aberdeen, Aberdeen, United Kingdom; 3 Alltech Biosciences Centre, Sarney, Summerhill Rd, Dunboyne, Country Meath, Ireland; National Cheng Kung University, TAIWAN

## Abstract

**Background:**

Selenium (Se) is an essential oligonutrient, as a component of several Se-containing proteins (selenoproteins), which exert important biological functions within an organism. In livestock, Se-enriched products have been proposed as dietary supplements to be included into functional feeds for animal preventive health care. To this end, it is important to understand the optimal range of concentrations for supplementation and how long it takes to be assimilated into the organism.

**Methods:**

In this study, rainbow trout (*Oncorhynchus mykiss*) were fed a control diet containing 0.9 g Kg^-1^ Se or the same diet supplemented with a Se-Yeast product (Sel-Plex) to achieve Se concentrations ranging from 1.5–8.9 g Kg^-1^ for a period of ten weeks. Fish were sampled every two weeks for analysis. The kinetics of Se bioaccumulation and the effects on fish selenoprotein expression was determined in different tissues combining chemical and bimolecular techniques.

**Results:**

The Sel-Plex enriched diets did not have any effect on survival and growth performance. The highest Se levels were found in liver and kidney followed by muscle and blood cells. Analysis of the Se concentration factor showed that liver is able to initially regulate the amount of Se accumulated. However, with higher dietary Se level (4.8 and 8.9 g Kg^-1^) and longer times of exposure (10 weeks), regulation is ineffective and the Se tissue concentration increases. The expression of the selected trout selenoprotein transcripts showed an inverse correlation with Sel-Plex augmentation in most cases. In liver, kidney and blood cells the highest up-regulation of the trout selenoprotein genes was seen mostly in the group fed the diet enriched with the lowest concentration of Sel-Plex (0.5 g Kg^-1^) for 10 weeks.

**Conclusion:**

Sel-Plex may represent an excellent Se supplement to deliver a high level of Se without provoking harm to the fish and to guarantee the maximal absorption of the element. According to our results, a dietary supplementation of Sel-Plex between 0.5 and 4 g Kg^-1^ may allow maximal benefits, whereas 8 g Kg^-1^ may be excessive for the purpose of supplementation.

## Introduction

Selenium (Se) is an essential element in nutrition, fundamental for the functioning of several Se containing proteins, called selenoproteins [1,2]. The number of selenoproteins (selenoproteome) may vary among different species [3]. Within vertebrates, 25 selenoproteins have been identified in mammals and up to 41 in bony fish (teleosts) [4]. Recent work has also shown that within teleosts, salmonid species may possess even more selenoproteins due to an additional whole genome duplication event that occurred during the evolution of this lineage, likely giving them one of the largest selenoproteomes known [5,6].

The function of several selenoproteins has been determined and found to be conserved across different species. Glutathione peroxidase (GPxs) and thioredoxin reductase (TrxRs) are the most studied and best characterized selenoproteins. They are an indispensable component of the cellular glutathione and thioredoxin systems respectively, and thus important regulators of the intracellular redox milieu [7,6]. Selenoprotein P (SelP) is the main protein within the organism responsible for Se homeostasis and transport through the body [8]. Interestingly SelP is present as two different isoforms in fish, SelPa and SelPb; they have marked differences at the structural level and likely have diverse roles in fish Se homeostasis [9]. Depending on their biological function, selenoproteins are commonly divided in two groups, essential and non-essential selenoproteins. The role of these proteins dictates a rigid hierarchical regulation of their synthesis, which becomes evident in conditions of low Se availability, where the expression of non-essential selenoproteins is impaired in favour of selenoproteins with essential functions [10,11].

Several biological processes rely on selenoproteins, most importantly for maintenance of antioxidant defenses, cell signaling, thyroid hormone metabolism and immune responses [9–13]. A severe Se deficiency can impair selenoprotein functioning and consequently can impact on an organism’s health status. Especially in a growing animal, it can result in several dysfunctions such as liver necrosis (in rats), pancreatic atrophy (in chicks), gizzard myopathy (in turkey poults), and “white muscle disease” (in sheep) [14]. In humans, some diseases are associated with Se deficiency (i.e. Keshan Disease, Kashin–Beck Disease and Myxedematous Endemic Cretinism) and their incidence is restricted to areas of the world particularly poor in Se [15]. However, too high a level of Se can be detrimental and can cause toxicity. In humans an excessive exposure to Se can induce selenosis, a state characterized by gastrointestinal disturbance, hair loss, fatigue, irritability, and mild nerve damage. In livestock, toxic levels of Se may cause alkali disease, which in the worst case scenario is lethal [16,17]. Among the essential elements, Se has a particularly narrow safety range between doses that lead to deficiency or toxicity [12,18], that complicates its use for supplementation purposes. Moreover, the recommended Se intake varies across species and according to geographic regions [19,20].

In livestock, Se-enriched products have been proposed either as a potential immunostimulant to be included into functional feeds for animal preventive health care, or as adjuvants to administer during prophylactic treatments. Hence, the potential of Se augmentation to improve the welfare of farmed animals is attracting a great deal of interest in livestock science and management, although there is still a lot of debate as to which Se concentrations should be considered. The physiological state of the organism can strongly affect the requirement for Se, and there may be great differences in requirement among different species. Studies across species can address the latter and in this context it is important to optimize clear biomarkers to monitor Se bioaccumulation at supranutritional levels, and to determine if Se is also being utilized and is biologically active within the organism [16].

The chemical form of Se delivered can strongly affect its bioavailability and therefore its impact on the organism. It is well established that inorganic forms of Se are less bioavailable than organic selenocompounds, and this means that inorganic Se can be more easily excreted rather than utilized for selenoprotein synthesis. Furthermore, inorganic selenocompounds exert toxicity at lower concentrations compared to organic forms [17]. Despite this, inorganic sodium selenite (Na_2_SeO_2_) has to date been the most studied and used Se supplement in nutritional sciences. It is a highly soluble salt, stable and easy to provide. Selenomethionine (Se-Met) is the preferred organic selenocompound for Se augmentation and is also the most abundant Se form found in food. Se-Met cannot be produced by animals, and it is synthesized in organisms such as fungi and plants, and then transferred along the food chain [21,22]. The concentrations of Se allowed to be used for human and livestock supplementation is governed by legislation and mainly based on studies carried out using Na_2_SeO_2_ and Se-Met. For aquaculture fish species the range of Se requirements has been set between 0.1 and 0.5 g Kg^-1^ (dry mass), and the amount of added Se to the diets cannot exceed 0.5 g Kg^-1^ (dry mass). However, in the past few years several more Se enriched products have been introduced to the market for Se supplementation. One of these, Se-Yeast, is a mixture of selenocompounds produced by yeast (mainly *Saccharomyces cerevisiae*) exposed to Na_2_SeO_2_, with Se-Met the most abundant selenocompound (representing roughly 70% of the total mass) in Se-Yeast based products [20]. The idea behind the commercialization of Se-Yeast is based on the fact that this compound can possibly deliver Se to consumers in a more natural form; moreover it is believed that selenocompounds in Se-Yeast are highly bioavailable and consequently it can be tolerated at higher concentrations [23].

In this study, we investigated the assimilation of the Se-Yeast product Sel-Plex in rainbow trout (*Oncorhynchus mykiss*) and its effect on the fish selenoproteome expression. Sel-Plex is a unique, standardized, registered food-grade Se-enriched yeast, manufactured by Alltech, with verified non-genotoxic effects on animals [24]. The use of natural and organic Se supplements such as Sel-Plex may represent a sustainable and environmentally friendly means to improve the health of farmed animals. To this end, it is important to understand the optimal range of concentrations for supplementation and how long it takes to be assimilated into the organism. Hence, in this experiment we fed rainbow trout a control diet containing 0.9 g Kg^-1^ Se or the same diet enriched with three different concentrations of Sel-Plex: 0.5, 4 and 8 g Kg^-1^, giving Se concentrations of 1.5 (low-Se diet), 4.8 (medium-Se diet) and 8.9 (high-Se diet) g Kg^-1^ respectively. The amounts of Sel-Plex added to the diets were chosen on the basis of previous published work [25]. During the feeding trial, the kinetics of bioaccumulation of Sel-Plex was measured in liver, blood, kidney and muscle by inductively coupled plasma mass spectrometry (ICP-MS). We also analysed the effect of Sel-Plex supplementation on selenoprotein biosynthesis in these tissues by measuring the expression, at the mRNA level by *q*PCR, of a number of newly characterized selenoprotein genes in this fish species.

## Materials and Methods

### Sel-Plex feeding trial design

All procedures were carried out under the UK Animals (Scientific Procedures) Act 1986 and Home office code of Practice guidance, under Home Office project licence PPL 60/4013. Rainbow trout (~100 g) were obtained from Almondbank (Perth, UK) and kept in 1-m-diameter fiberglass tanks supplied with recirculating freshwater at 15 ± 1°C, containing 50 mg/l of dissolved oxygen, within the aquarium facilities in the School of Biological Sciences, University of Aberdeen. For the feeding trial, 200 fish were distributed in eight tanks; two tanks were assigned to each diet, giving 50 fish per diet group. After an acclimatization period of four weeks, a ten week feeding trial was carried out. Four diets were used: the acclimatization diet as control and three diets enriched with different concentrations of Sel-Plex as experimental diets. All the diets were prepared by the Hellenic Centre for Marine Research, Greece (HCMR, www.hcmr.gr) and the composition is described in Table 1. Briefly, the vitamins and Sel-Plex were stirred in a small mixer (Kenwood KM260), whilst the remaining raw materials (excluding the fish oil) were placed in a large mixer (Hobart A200 DT). After mixing for 15 min, the two components were combined together in the large mixer for an additional 15 min before adding the fish oil, and mixing for a further 15 min. The mixture was extruded (Clextral EV0 A107) at 50°C, 70°C, 80°C, 91°C and 100°C, in five consecutive barrels. The pellets obtained were dried O/N at 35°C in a forced air circulator.

**Table 1:** Dietary formulations of the experimental diets.

	Acclimatization diet	Low-Se	Medium-Se	High-Se
	Control diet	Diet	Diet	Diet
*Fish meal*	150	150	150	150
*Wheat meal*	200	199.5	196	192
*Corn gluten*	190	190	190	190
*Soya*	160	160	160	160
*Fish oil*	165	165	165	165
*Wheat gluten*	130	130	130	130
*Vitamins*	5	5	5	5
*Sel-Plex*	-	0.5	4	8

All the values are in g Kg^-1^.

The Se availability from each diet was analysed and fish were fed twice daily an amount equal to 2% of their average body weight. Six fish per tank were weighed at the beginning of the feeding trial, and subsequently every two weeks until the end of the trial, and the amount of feed given adjusted accordingly. Specific growth rate (SGR) was calculated using the following formula:

$$\text{SGR}\;\left(\%\;{\text{increase body wt d}}^{-1}\right)=\left[\left(\ln_{\text{W}2}–\ln_{\text{W}1}\right)/\text{days}\right]\times 100;$$

W1=start weight(g),W2=final weight(g),days=days in the growth period

### Sample preparation

After two, four, six, eight and ten weeks from the beginning of the feeding trial three fish from each tank (i.e. 6 fish per diet) were killed for tissue harvest. Animals were anaesthetized in 2-phenoxyethanol (Sigma-Aldrich), blood was collected from the caudal vein and afterwards the fish were killed and muscle, liver and kidney were sampled for biological and chemical analysis. Blood was sampled to examine gene expression in blood cells, primarily composed of erythrocytes, which in teleost fish are nucleated cells and have the machinery necessary for RNA transcription, post-transcriptional modifications and protein translation [26,27].

The blood, muscle, liver and kidney samples were processed to measure Se bioaccumulation. Tissues from the first (after two weeks), third (after six weeks) and fifth (after ten weeks) time points were processed for transcript expression analysis. For RNA extraction, 100 mg of muscle, liver and kidney were first placed on dry ice, to which 1.4 ml of TRIzol (Sigma-Aldrich) was added. A sub-aliquot of blood of 100 μl was diluted 1:10 in Hank's Balanced Salt Solution (HBSS, Gibco) and centrifuged at 1000 rpm for 2 min. The blood cell pellet was then dissolved in 1.4 ml of TRIZol. All the samples were then homogenized using a tungsten carbide bead (5 mm, Qiagen) and the TissueLyser II system (Qiagen), at 30 Hz for 3 min, and further processed for cDNA synthesis as described in section 3.4. The rest of the muscle, liver, kidney and the whole blood samples were freeze- dried, ground using a mortar and pestle and subsequently processed for analysis of Se bioaccumulation (described in section 3.3).

### Sample decomposition for total Se analysis

For total Se analysis of the diets and trout tissues, 100 mg of the lyophilized pellets/organs (three replicates of each) were weighed into a 50 ml Teflon vessel. Nitric acid (1 ml) was added to each vessel containing the samples and allowed to stand overnight. Hydrogen peroxide (2 ml) was then added to the vessel, which was closed to prevent analyte loss. The samples were digested using a CEM mars microwave digester (CEM MARXPRESS). The samples were brought to 20 ml and analysed using an ICP-MS (Agilent 7500 series). The ICP-MS was operated with a forward power of 1380 W under normal conditions with a nickel sampler and skimmer cones. Carrier gas flow was 1.27 L/min, coolant gas flow 14 L/min and nebuliser gas flow 0.86 L/min. Total Se concentration was determined by monitoring ^77^Se and ^78^Se isotopes by external calibration. Germanium was used as an internal standard. In order to evaluate the accuracy of the method total selenium content in dogfish (*Sycliorhinus canicula*) muscle (DORM—2) was measured at the end of each time point analysis and the percentage recovery range determined was between 99.5–102%.

The Concentration factor (CF), the ratio between the concentration of a chemical in an organism (or a tissue) and the concentration of the chemical in the feed, was calculated for each tissue using the following formula:

CF=(Concentration in tissue)/(Concentration in feed)

### Selenoprotein expression analysis

Tissues were homogenised in TRIzol (Sigma), with 5mm stainless steel beads (Qiagen) and shaken using the TissueLyser II system, at 15 Hz for 2 min. RNA was extracted by adding 300 μl of chloroform to the lysates. After 5 min incubation on ice, samples were centrifuged at 15,700 × g for 15 min at 4°C for complete separation of the mixture. The colorless upper aqueous phase containing RNA was transferred to a fresh RNase-free 1.5 ml tube containing an equal amount of chilled isopropanol (Sigma), and the RNA was subsequently precipitated at -80°C. To collect the RNA precipitates, the supernatant was removed after centrifugation at 15,700 × g for 10 min at 4°C and the pellet washed with 70% ethanol, air dried and resuspended in RNase free H_2_O. The concentration and purity was determined by spectrophotometry (Nanodrop). The first strand cDNA was synthesized from 2 μg of total RNA using 1 μl RevertAid reverse transcriptase (10,000 U, Fermentas) in the presence of 5 μl 5×Reaction Buffer, 1 μl dNTP (Bioline), made up to a final volume of 25 μl with water and incubated at 42°C for 2 h.

QPCR was performed with a LightCycler 480 (Roche) to quantify the expression of selected selenoprotein transcripts and the common reference genes using the primers given in Table 2. The primers employed for *q*PCR were designed with at least one primer across a predicted intron and pre-tested to ensure that each primer pair could not amplify genomic DNA using the *q*PCR protocols. The *q*PCRs were performed in duplicate for each sample, along with a 10-fold serial dilution of references consisting of an equimolar mix of purified PCR products of each gene amplified from cDNA. The transcript level was calculated using the quantitative fit points method in the integrated LightCycler 480 software. In this study we screened for the transcript expression of the different isoforms of trout GPx1 and TrxR3 genes characterized in previous studies [5,6], and the isoforms for trout *selpa* and *selpb* transcripts recently identified in our lab (Acc. No. HF969249 and HF969250). The relative expression level of each mRNA in the different tissues was expressed as arbitrary units that were calculated from the serial dilution of references run in the same 384-well plate and normalized against the geometric mean of the expression level of elongation factor 1 *α* (*ef-1α)*, DNA directed RNA polymerase III subunit I *(drpII)* and hypoxanthine phosphoribosyltransferase 1 (*hprt1*). Fold changes were also calculated as the average expression level of each treatment group divided by that of the corresponding controls.

### Statistical analysis

Data are presented as means ± S.E.M. plotted using GraphPadPrism software (V5), and analysed using the SPSS package 18.0 (SPSS Inc. Chicago, Illinois). All data were analysed using one way-analysis of variance (ANOVA) and Tukey's post hoc test to evaluate whether the means were significantly different among the groups. Significant differences were indicated at *p* < 0.05. Prior to one-way ANOVA, data were square root transformed to meet ANOVA assumptions of normality and homogeneity of variance.

**Table 2:** Primers used for *q*PCR.

Gene name	Primer	Primer sequence	Product	Acc
	name	(5’ → 3’)	size (bp)	N°
Selenoprotein Pa	SelPa-F	GCTTGGTGCAGGCATCCTTATTG	276	HF969249
	SelPa-R	CATATCTCCCTGCCCTACTCCATCC		
Selenoprotein Pb	SelPb-F	GACGACTTCCTGGTATATGACAGATGTG	275	HF969250
	SelPb-R	GATACCGTCAGCAACCCAGTTCC		
Thioredoxin reductase 3a	TrxR3a-F	AGTCAACCCCAAGAACGGTAAGG	297	HF969246
	TrxR3a-R	CAGAAGAGACTGTGGTACACCTCCAA		
Thioredoxin reductase 3b	TrxR3b-F	CAAAGTCAACCCCAAGAATGGTAAGA	300	HF969247
	TrxR3b-R	CAGAAGAGACTGTGGTACACCTCCAG		
Glutathione peroxidase 1a	GPx1a-F	ATGAAATGGCTGGGAAAATAAAGA	250	HE687021
	GPx1a-R	TCATCATTCTTACAATTCTCCTGATG		
Glutathione peroxidase 1b1	GPx1b1-F	CAACATGTCTGGAAGTGAGTTCTACAACA	241	HE687022
	GPx1b1-R	TTCGTTATTGCAGTTCTCCTGATGTC		
Glutathione peroxidase 1b2	GPx1b2-F	ACCAGGCAAATGGCTGTATGTAAGAT	250	HE687023
	GPx1b2-R	CTTCGTTCTTGCAGTTCTCCTGATG		
Elongation factor-1α	EF1α-F	CAAGGATA-TCCGTCGTCGTGGCA	327	AF498320
	EF1α-R	ACAGCGAAACGACCAAGAG		
DNA directed RNA	DRPII-F	TCACCCATGAAGTTGATGAGCTGA	176	BT073753
polymerase III subunit I	DRPII-R	CCGTGCAGACATAGTACAGCCTCA		
Hypoxanthine	HPRT1-F	GCCTCAAGAGCTACTGCAATG	256	ACH70616
Phosphoribosyltransferase 1	HPRT1-R	GTCTGGAACCTCAAATCCTATG		

## Results

### Growth rate and feed performance

**Fig 1:**
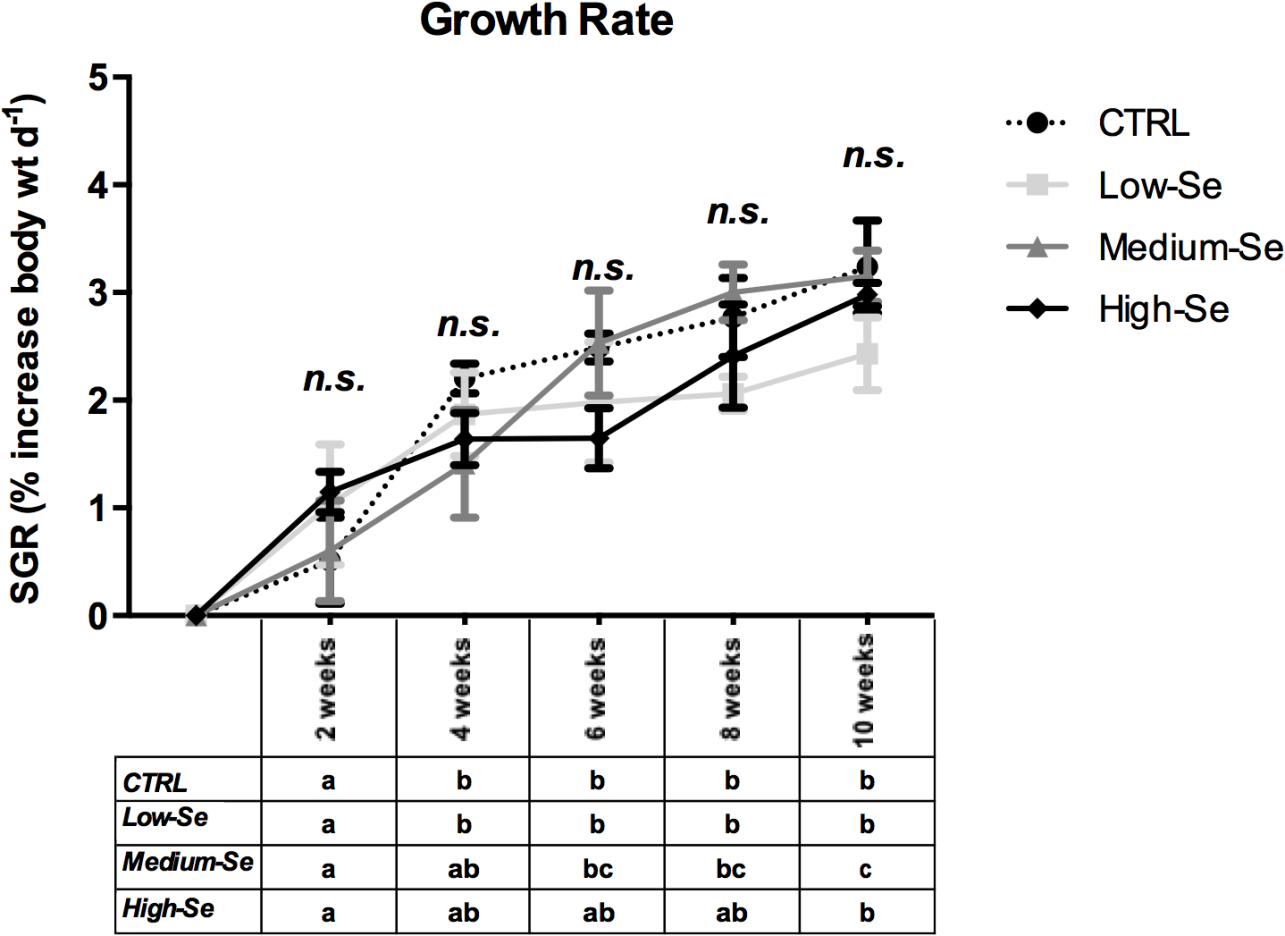
Growth performance of rainbow trout given various levels of organic supplemental Se for 10 weeks. The results represent the mean **±** SEM from six fish per diet at each time point. On the graph the significance of the difference in body weight between the diets groups at each time point is indicated, whilst in the table underneath the changes in body weight for each diet group across the different time points is reported. Values not statically different are indicated by “n.s.”, whereas values significantly different (*p<*0.05) are indicated by different letters.

A feeding trial using Sel-Plex supplemented diets was carried out as described in section 3.1. Growth rates of the fish in the diet trial were within the normal range for rainbow trout raised under production conditions and supplemental Se had no effect on SGR (Fig 1), in agreement with previous studies on Se supplementation in trout [25]. During the 10-week period the fish increased in weight from 88.85 ± 9.76 g to 232.67 ± 9.62 g.

### Selenium bioaccumulation and bioconcentration

Total selenium concentration was measured in all the diets used in this feeding trial (Table 3), to verify the Se background coming from the raw material (*e*.*g*. fish meal and fish oil) and to verify the Sel-Plex supplementation.

**Table 3:** Se concentration in the feed pellets of the four different diets used for this feeding trial.

	Total Se	Se-Met
	concentration	concentration
	(g Kg^-1^) ± SEM	(g Kg^-1^) ± SEM
*Acclimatization diet/Control diet*	0.87 ± 0.018	0.75 ± 0.12
*Low-Se*	1.46 ± 0.01	1.40 ± 0.09
*Medium-Se*	4.81 ± 0.075	4.46 ± 0.16
*High-Se*	8.94 ± 0.063	8.20 ± 0.24

The results represent the mean and SEM of four independent measurements.

**Fig 2:**
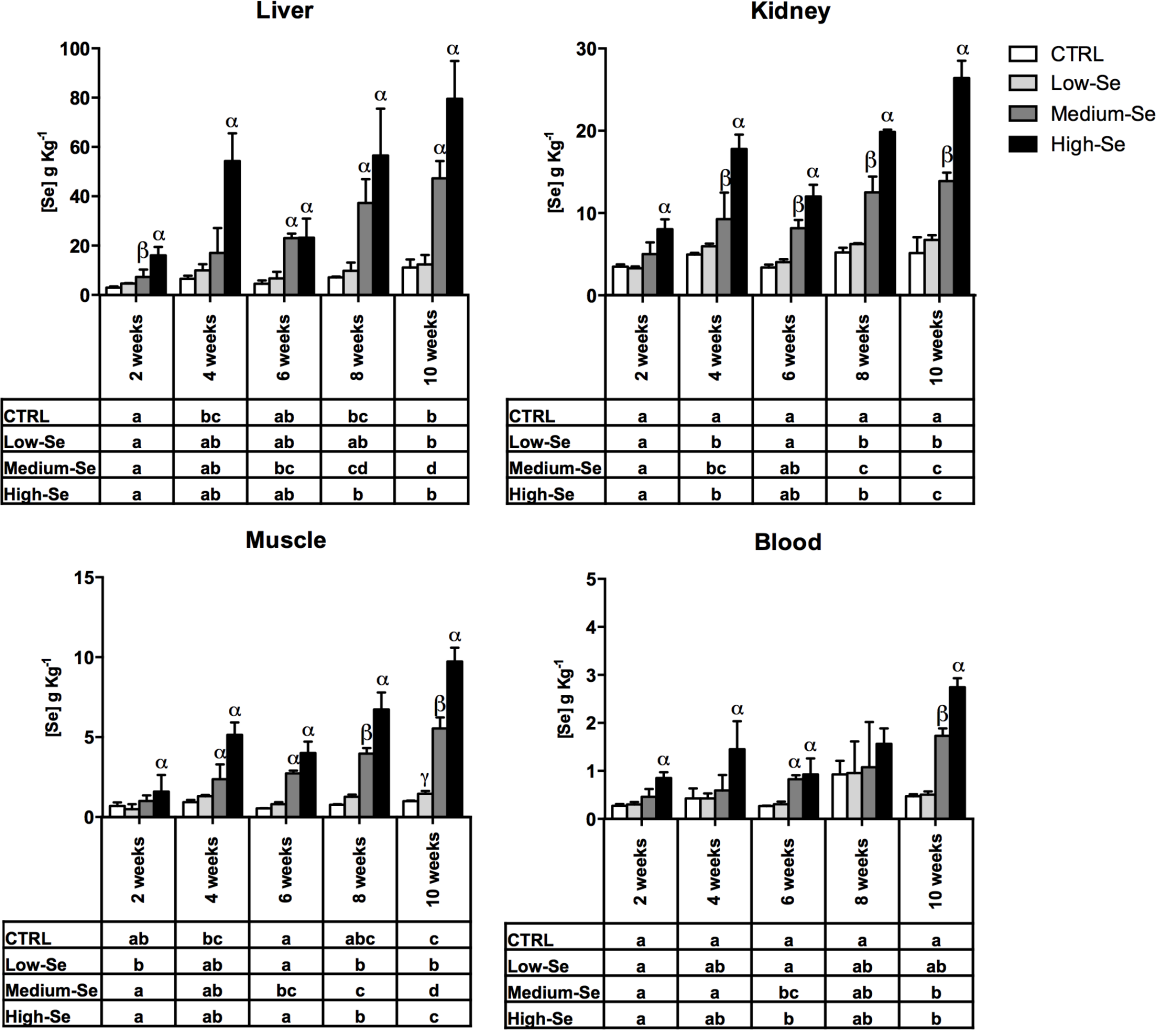
Tissue specific assimilation of Se in rainbow trout fed a control diet and three diets enriched with different concentrations (0.5, 4 and 8 g Kg^-1^) of Sel-Plex for ten weeks. The results represent the mean + SEM of four independent measurements. The Greek letters above the columns indicate values statistically significant from the controls (*p*<0.05), with different letters indicating significant differences between the groups fed the Sel-Plex supplemented diet at each time point. The tables below the graphs indicate differences in Se level within the same diet group across the different time points.

Fish bioaccumulation of Se was measured in liver, kidney, muscle and blood cells of fish fed the control diet or diets enriched with different concentrations of Sel-Plex (0.5, 4 and 8 g Kg^-1^), since these organs are the main sites of Se bioaccumulation [28]. In all the organs targeted in this study there was a relative and proportional increase in tissue Se concentration to dietary Se concentration with time (Fig 2). The highest concentration was recorded in the liver followed by the kidney, muscle and blood.

In the liver tissue no significant difference in the bioaccumulation of Se between the control group and the fish fed the low-Se enriched diet was seen. In contrast, in the groups fed the medium- and high-Se enriched diets, a significant increment in Se bioaccumulation was seen after two weeks feeding. In the group fed the diet enriched with the highest concentration of Sel-Plex, the level reached after four weeks was comparable with the amount of Se measured in the same group after 10 weeks (as shown in Fig 2).

The kidney showed a similar trend in Se bioaccumulation but had a lower total concentration. In the kidney, the group fed the high-Se enriched diet was the only one that had a significant difference from the control group after two weeks of feeding. The group fed the medium-Se enriched diet showed a significant bioaccumulation starting after 4 weeks of feeding, whereas no differences from the control were recorded in the group fed the low-Se enriched diet.

In the muscle, a similar trend in Se bioaccumulation was seen compared to the liver and kidney. In this tissue, accumulation of Se was seen at the first time point only in the group fed the high-Se enriched diet. This group also had a significantly higher concentration of Se bioaccumulation compared to the group fed the medium-Se enriched diet after 10 weeks of feeding. Interestingly, in muscle there was a small but significant bioaccumulation detected in the group fed the low-Se enriched diet in contrast to the other tissues.

The Se concentration in blood cells was the lowest recorded. Only in the group fed the highest concentration of Sel-Plex was a correspondence between the accumulation of Se in blood cells and the other three tissues found. However, at week 8 no significant difference was seen primarily due to a larger than normal variation present in these samples at this time.

In kidney and muscle, a plateau in Se bioaccumulation was not reached, most evident in the group fed the high-Se enriched diet where a significant increment in Se tissue concentration was seen at 10 weeks versus 8 weeks of the feeding trial. Overall, the data show a constant increase in Se bioaccumulation, suggesting that prolonging the feeding could have allowed even higher tissue concentrations of Se.

**Fig 3:**
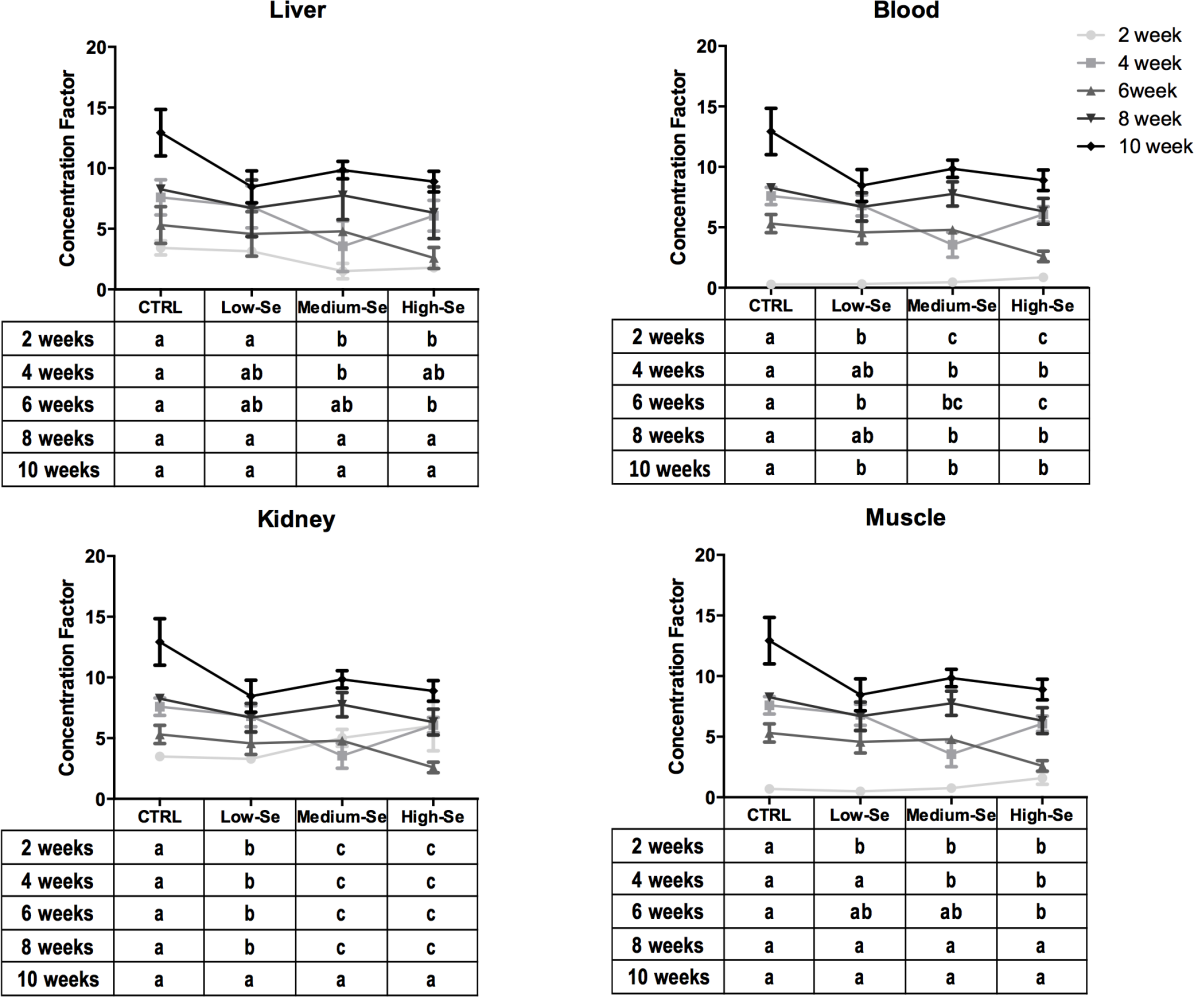
Concentration factor of Se in rainbow trout fed a control diet and three diets enriched with different concentrations (0.5, 4 and 8 g Kg^-1^) of Sel-Plex for ten weeks. The results represent the mean ± SEM of four independent measurements. The letters above the columns indicate values statistically significant from the controls (*p*<0.05), with different letters indicating significant differences between the treatments. The tables below the graphs indicate differences in Se levels within the same diet group across the different time points.

Concentration factor (CF) was used as a relative index to illustrate how selenium is handled physiologically by the different fish tissues at the different concentrations of Sel-Plex tested in this study (Fig 3). The CF is high at lower Se levels (control diet) and decreases as Se level increases under normal homeostasis [29]. A decrease in the CF with increased dietary Se concentration indicates regulation of selenium uptake by tissues, whereas an increase (or resistance) in CF with increased dietary Se concentration would indicate a poor regulation and consequent Se retention.

After two weeks of feeding, the CF of liver was inversely correlated with the level of dietary Se, whereas muscle, kidney and blood CF increased with dietary concentration of Se. Thus the liver behaves differently from the other tissues and appears to initially regulate the concentration of Se accumulated. After four weeks, the CF in all the tissues decreased with dietary Se concentration, except for the group fed the high-Se enriched diet suggesting that at a dietary concentration lower than 8.9 g Kg^-1^ all the tissues are able to regulate Se bioaccumulation.

From 6 weeks of feeding onwards, Se accumulated in all the tissues of the groups fed the medium- and high-Se enriched diets. At such high Se concentrations and after a longer exposure time, regulation likely becomes less effective and the metal burden increases. Usually, this effect is associated with toxic metals, with the potential that Se at relatively high concentrations can be harmful to the fish. However during the feeding trial, fish did not manifest any signs of toxicity, in agreement with the proposed capability of aquatic organisms to regulate Se internally and minimize bioaccumulation at key sites.

### Analysis of trout selenoprotein transcripts in fish fed diets supplemented with Sel-Plex

**Fig 4:**
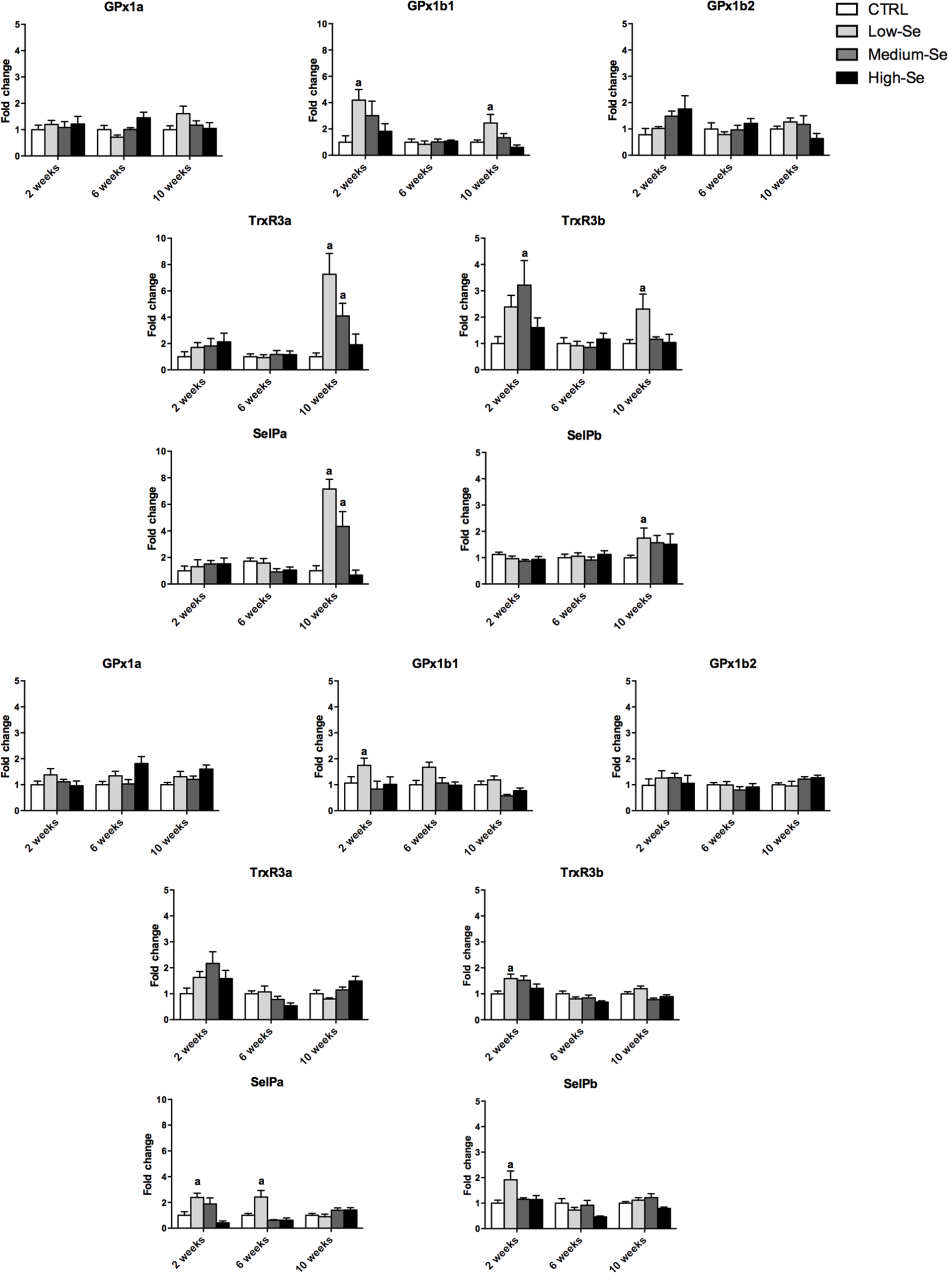
Transcriptional modulation of trout *gpx1*, *trxr3*, and *selp* isoforms in liver (A) and kidney (B) from rainbow trout fed a control diet and three diets enriched with different concentrations (0.5, 4 and 8 g Kg^-1^) of Sel-Plex for 2, 6 and 10 weeks. The expression of gene transcripts was quantified by *q*PCR and normalized against the geometric mean of three housekeeping genes (*ef-1α*, *drpII*, *hprt*) from the same sample, and then used for statistical analysis. A fold change, calculated as the average expression level of stimulated samples divided by that of the controls, is presented. The results represent the mean + SEM from six fish. The letters above the columns indicate values statistically significant from the controls (*p<*0.05), with different letters indicating significant differences between the treatments.

**Fig 5:**
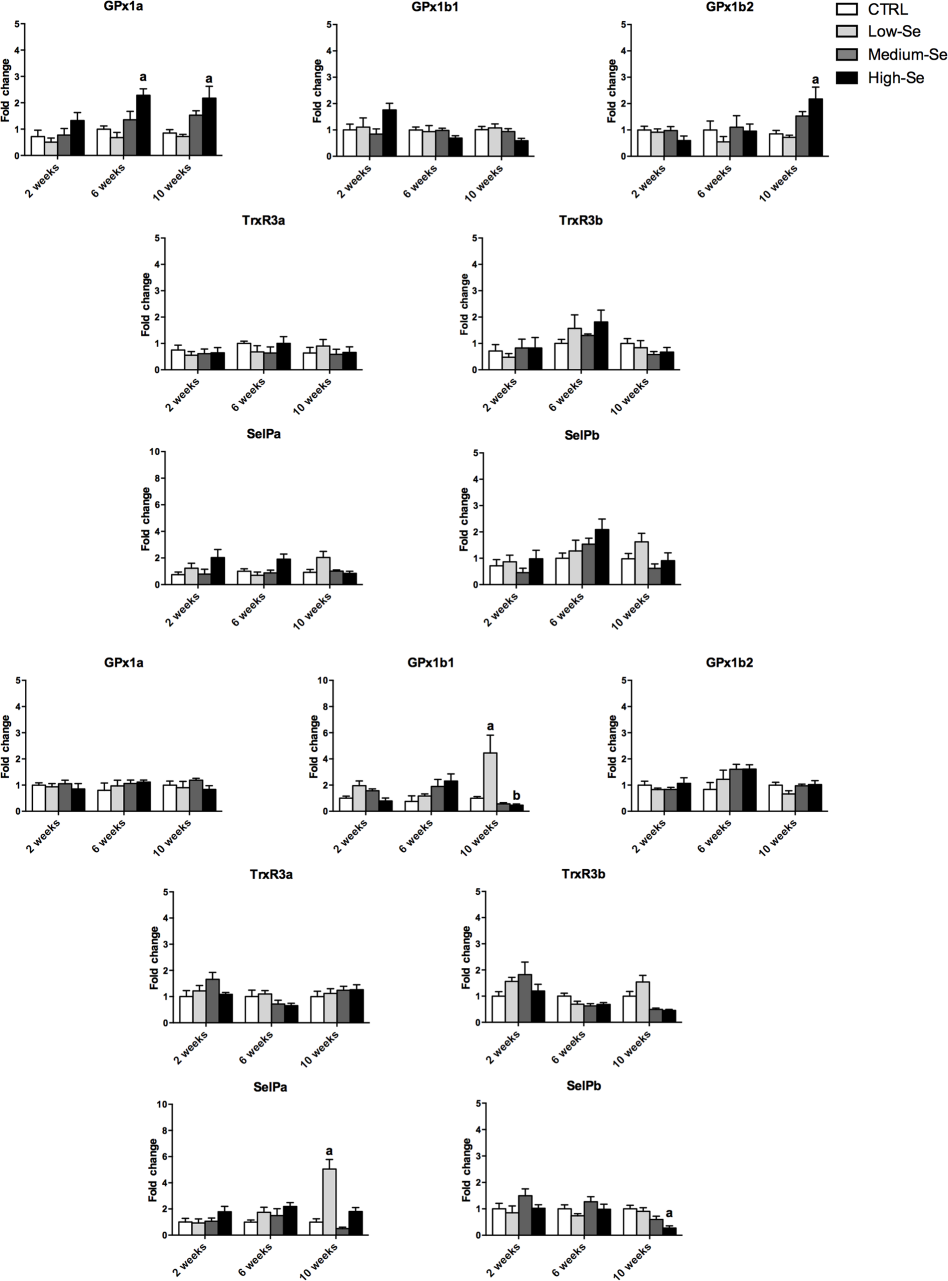
Transcriptional modulation of trout *gpx1*, *trxr3*, and *selp* isoforms in blood cells (A) and muscle (B) from rainbow trout fed a control diet and three diets enriched with different concentrations (0.5, 4 and 8 g Kg^-1^) of Sel-Plex for 2, 6 and 10 weeks. The expression of gene transcripts was quantified by *q*PCR and normalized against the geometric mean of three housekeeping genes (*ef-1α*, *drpII*, *hprt*) from the same sample, and then used for statistical analysis. A fold change, calculated as the average expression level of stimulated samples divided by that of the controls, is presented. The results represent the mean + SEM from six fish. The letters above the columns indicate values statistically significant from the controls (*p<*0.05), with different letters indicating significant differences between the treatments.

Modulated expression of selected trout selenoprotein transcripts was detected in all the fish tissues taken for analysis in this study (liver, blood cells, kidney and muscle). The increases seen ranged from 1.5 to 10 fold in the groups fed diets enriched with the different concentrations of Sel-Plex compared to the control diet group (Figs 4 and 5).

The liver was the most responsive tissue at the transcriptomic level, followed by kidney, blood cells and muscle. This directly reflects the accumulation of Se in these tissues described earlier and further confirms the importance, especially of liver and kidney, in Se metabolism within fish. Interestingly in liver, kidney and blood cells the highest response in selenoprotein transcript expression was seen in the fish fed the low-Se enriched diet, followed by the medium-Se enriched diet group, and mainly at the last time point (10 weeks) in liver and blood cells. Five of the seven genes studied were modulated in the liver at week 10, with only GPx1a and GPx1b2 unaffected. Similarly, GPx1b1 and SelPa were upregulated in blood cells at this time.

In the kidney the response to the low-Se enriched diet was mainly seen at week 2 in this tissue, with GPx1b1, TrxR3b, SelPa and SelPb all upregulated to some degree. This contrasts to the response in muscle where a significant increase in GPx1a and GPx1b2 was seen in fish fed the high-Se enriched diet for 10 weeks; genes that were not affected in the other tissues. Only in the blood cells was a significant inhibition of selenoprotein transcript expression detected, of GPx1b1 and SelPb, also at week 10. In the same tissue and time point, a slight but significant reduction of GPx1b1 and SelPb transcripts was seen, likely due to detrimental effects of the high Se levels on this specific tissue.

## Discussion

Selenium (Se) is an essential element in human and animal diets [30,31]. Its deficiency has various negative impacts but the line between Se requirement and toxicity can be narrow [32]. The low level of Se in many countries has been of increasing interest in the supplementation of human food and animal feed with this element [23]. The Se requirement across species may vary considerably, but to date no studies have addressed this to any extent. Previous work has reported a range of the Se requirement in fish between 0.1 and 0.5 g Kg^-1^ (dry mass) [33,34]. Up to 3 g Kg^-1^ rainbow trout can regulate Se accumulation through excretion but over this level Se rapidly bioaccumulates and starts to exert detrimental effects on fish, with elevated mortality seen when using over 13 [35]. In line with these studies, the European Union has legislated that, if additional Se is to be added into the feed composition, the total feed level must not exceed 0.5 g Kg^-1^ (dry mass) (Commission Regulation EU No 432/2012). However, more recent investigations have provided evidence that farmed fish may require a higher content of Se in their diet, and if it is delivered in an organic form it may be more digestible, better accumulated in tissue, and more biologically active [36–38]. There is also evidence that the Se requirement in fish subjected to stressful conditions may be up to 4.0 g Kg^-1^ (dry mass) [25,39].

Nutritionally complete diets are necessary in culture situations, therefore micronutrients must be supplied at adequate levels in the prepared diets to support optimal growth and production efficiency. This is particularly true in intensive situations where immune competence and disease resistance can be compromised by deficiencies of various nutrients and crowded conditions. Dietary supplementation of some of these micronutrients in excess of the minimum requirement levels has been shown to significantly enhance the immune response and disease resistance of various animals [40]. An effective and relatively inexpensive solution for Se supplementation is the use of refined products from yeast *(S*. *cerevisiae*) enriched in Se [41].

In this study we have measured the bioaccumulation of Se in different tissues key for Se metabolism in rainbow trout, following dietary supplementation with Sel-Plex, during a time course feeding trial. The fish tolerated all the Sel-Plex/Se concentrations tested (0/0.9, 0.5/1.5, 4/4.8 and 8/8.9 g Kg^-1^), without showing external signs of toxicity. No effects on growth rate were seen, in agreement with what is already reported in the literature [25].

Liver and kidney showed the highest accumulation of Se, likely due to their role as scavenging and clearance organs. Looking at liver Se bioaccumulation, the group fed the highest Sel-Plex concentration reached a plateau after 4 weeks with levels of Se comparable with the concentrations detected in the same group at the end of the feeding trial (10 weeks). Instead, in the kidney and muscle a plateau in Se bioaccumulation was not reached, most evident in the groups fed medium- and high-Se enriched diets for 8 and 10 weeks. In both tissues, no significant differences were detected in the group fed the low-Se enriched diet compared to the control group. In contrast, in the muscle a significant bioaccumulation of Se was seen in the group fed the low-Se enriched diet after 10 weeks of feeding, suggesting a higher retention rate for this tissue. The levels found in the muscle were relatively low compared to liver and kidney; however this tissue represents approximately 70% of the total fish mass, therefore the Se absorbed from the diet may have been diluted within the entire muscular mass. The bioaccumulation of Se in the fish fillet is extremely important because of its potential impact on the consumer. Fish diets enriched with Se-Yeast based supplements may represent an efficient alternative to deliver Se enriched food, especially for countries with a deficiency of this element. Moreover, Se supplementation in yeast form appears to be bioavailable for fish and well tolerated, highlighting the advantage in using a yeast-derived product for Se augmentation.

When considering the CF, it increased with dietary Se level in all the tissues, with the exception of the group fed the high-Se enriched diet where the accumulation level dropped. This suggests that at these dietary concentrations and times trout cannot regulate the excess selenium which might subsequently lead to detrimental effects.

To investigate the biological response to Sel-Plex supplementation we next examined the effect of Sel-Plex supplementation on the mRNA expression of selected trout selenoproteins. Whilst it is not possible to rule out the potential effects of the yeast component in Sel-Plex on gene expression, the data clearly reflect the overall impact of the commercial supplement. Study of the expression of the selenoprotein transcripts showed an inverse correlation with Sel-Plex augmentation in most tissues. In liver and blood cells the largest up-regulation of the trout selenoprotein genes studied was mostly in the group fed the low-Se enriched diet for 10 weeks, especially for the *gpx1b1* transcript and the isoforms of TrxR3 and SelP. Only in muscle was a significant induction of the *gpx1a* and *gpx1b2* transcripts detected, and this was in the group fed the high-Se enriched diet. The expression of these two *gpx* isoforms at the highest Sel-Plex concentration in the muscle could be associated with the occurrence of oxidative stress due to the potential pro-oxidant effects of Se at high concentrations. The differential expression pattern of the GPx gene isoforms is in accord with their phylogenetic relationship. Indeed, as discussed previously [5] *gpx1a* and *gpx1b2* isoforms likely result from the duplication of the GPx1 gene, whereas trout *gpx1b1* is more related to a form known in mammals as the gastro-intestinal specific GPx (GPx2). In addition, a slight but significant inhibition was seen for the *gpx1b1* and *selpb* transcripts in blood cells from fish fed the high-Se enriched diet. It is possible that this specific tissue is more susceptible to the detrimental effects of high Se concentration, and the transcription of these two specific genes became compromised, perhaps because they are not essential for cell survival. Collectively these data support the possibility that low levels of Sel-Plex supplementation, in the range of 0.5 to 4 g Kg^-1^ may be more beneficial to improve selenoprotein expression, with the effects seen using 8 g Kg^-1^ potentially reflecting an inability to effectively regulate Se bioaccumulation. However, measurement of markers of oxidative damage, such as DNA degradation, lipid peroxidation, glutathione reduction and total scavenging capacity, are needed to verify the potential beneficial or detrimental effects of Se supplementation.

In conclusion, the results of the present study suggest that Sel-Plex may represent an excellent Se supplement to deliver a high level of Se without provoking harm to the fish and to guarantee the maximal absorption of the element within tissues. A concentration of 0.5 g Kg^-1^ Sel-Plex may be already sufficient to guarantee Se enrichment in the fish fillet, with possible benefits for human consumption. According to our chemical and biological results, 8 g Kg^-1^ Sel-Plex may be excessive for the purpose of supplementation, whereas a dietary concentration of between 0.5 and 4 g Kg^-1^, with the caveat that this study was limited to 10 weeks, appears to represent a range that may allow maximal benefits.

## References

[1] Papp LV, Lu J, Holmgren A, Khanna KK. (2007) From selenium to selenoproteins: Synthesis, identity, and their role in human health. Antioxidants and Redox Signaling 9: 775–806. 17508906 10.1089/ars.2007.1528

[2] Hatfield DL. (2002) How selenium has altered our understanding of the genetic code. Mol Cell Biol 22: 3565 11997494 10.1128/MCB.22.11.3565-3576.2002 PMC133838

[3] Lobanov AV, Hatfield DL, Gladyshev VN. (2009) Eukaryotic selenoproteins and selenoproteomes. Biochimica et Biophysica Acta—General Subjects 1790: 1424–1428. 10.1016/j.bbagen.2009.05.014 PMC3471088 19477234

[4] Mariotti M, Ridge PG, Zhang Y, Lobanov AV, Pringle TH, Guigo R, et al (2012) Composition and evolution of the vertebrate and mammalian selenoproteomes. PLoS ONE 7: e33066 10.1371/journal.pone.0033066 22479358 PMC3316567

[5] Pacitti D, Wang T, Page MM, Martin SAM, Sweetman J, Feldmann J, et al (2013) Characterization of cytosolic glutathione peroxidase and phospholipid-hydroperoxide glutathione peroxidase genes in rainbow trout (*Oncorhynchus mykiss*) and their modulation by in vitro selenium exposure. Aquatic Toxicology 130–131: 97–111. 10.1016/j.aquatox.2012.12.020 23384997

[6] Pacitti D, Wang T, Martin SAM, Sweetman J, Secombes CJ. (2014) Insights into the fish thioredoxin system: Expression profile of thioredoxin and thioredoxin reductase in rainbow trout (*Oncorhynchus mykiss*) during infection and in vitro stimulation. Developmental & Comparative Immunology 42: 261–277. 24095766 10.1016/j.dci.2013.09.013

[7] Hawkes WC, Alkan Z. (2010) Regulation of redox signaling by selenoproteins. Biol Trace Elem Res 134: 235–251. 10.1007/s12011-010-8656-7 20306235 PMC2855032

[8] Köhrle J, Schweizer U, Schomburg L. (2012) Selenium transport in mammals: Selenoprotein P and its receptors In: Hatfield DL, BerryVadim MJ, Gladyshev VN, editors. Selenium: Springer pp. 205–219.

[9] Zavacki AM, Marsili A, Larsen PR. (2012) Control of thyroid hormone activation and inactivation by the iodothyronine deiodinase family of selenoenzymes In: Hatfield DL, BerryVadim MJ, Gladyshev VN, editors. Selenium: Springer pp. 369–381.

[10] Huang Z, Rose AH, Hoffmann PR. (2012) The role of selenium in inflammation and immunity: From molecular mechanisms to therapeutic opportunities. Antioxidants and Redox Signaling 16: 705–743. 10.1089/ars.2011.4145 21955027 PMC3277928

[11] McKenzie RC, Arthur JR, Beckett GJ. (2002) Selenium and the regulation of cell signaling, growth, and survival: Molecular and mechanistic aspects. Antioxidants and Redox Signaling 4: 339–351. 12006185 10.1089/152308602753666398

[12] Tapiero H, Townsend DM, Tew KD. (2003) The antioxidant role of selenium and seleno-compounds. Biomedicine and Pharmacotherapy 57: 134–144. 12818475 10.1016/s0753-3322(03)00035-0 PMC6361120

[13] Rayman MP. (2012) Selenium and human health. The Lancet 379: 1256–1268. 10.1016/S0140-6736(11)61452-9 22381456

[14] Hefnawy AEG, Tórtora-Pérez JL. (2010) The importance of selenium and the effects of its deficiency in animal health. Small Ruminant Research 89: 185–192.

[15] Combs GF Jr. (2001) Selenium in global food systems. Br J Nutr 85: 517–547. 11348568 10.1079/bjn2000280

[16] Sunde RA. (2012) Selenoproteins: Hierarchy, requirements, and biomarkers In: Hatfield DL, BerryVadim MJ, Gladyshev VN, editors. Selenium: Springer pp. 137–152.

[17] Thiry C, Ruttens A, De Temmerman L, Schneider Y, Pussemier L. (2012) Current knowledge in species-related bioavailability of selenium in food. Food Chem 130: 767–784.

[18] Spallholz JE. (1997) Free radical generation by selenium compounds and their prooxidant toxicity. Biomedical and environmental sciences 10: 260 9315319

[19] Kieliszek M, Błażejak S. (2013) Selenium: Significance, and outlook for supplementation. Nutrition 29: 713–718. 10.1016/j.nut.2012.11.012 23422539

[20] Bierla K, Szpunar J, Yiannikouris A, Lobinski R. (2012) Comprehensive speciation of selenium in selenium-rich yeast. TrAC Trends in Analytical Chemistry 41: 122–132.

[21] Dumont E, Vanhaecke F, Cornelis R. (2006) Selenium speciation from food source to metabolites: A critical review. Analytical and Bioanalytical Chemistry 385: 1304–1323. 16830114 10.1007/s00216-006-0529-8

[22] Fairweather Tait SJ. (1999) The importance of trace element speciation in nutritional sciences. Fresenius J Anal Chem 363: 536.

[23] Rayman MP. (2004) The use of high-selenium yeast to raise selenium status: How does it measure up? Br J Nutr 92: 557–573. 15522125 10.1079/bjn20041251

[24] Griffiths JC, Matulka RA, Power R. (2006) Genotoxicity studies on Sel-Plex, a standardized, registered high-selenium yeast. Int J Toxicol 25: 477–485. 17132606 10.1080/10915810600959667

[25] Rider SA, Davies SJ, Jha AN, Fisher AA, Knight J, Sweetman JW. (2009) Supra-nutritional dietary intake of selenite and selenium yeast in normal and stressed rainbow trout (*Oncorhynchus mykiss*): Implications on selenium status and health responses. Aquaculture 295: 282–291.

[26] Morera D, MacKenzie SA. (2011) Is there a direct role for erythrocytes in the immune response? Vet Res 42. 10.1186/1297-9716-42-89 PMC3199785 21801407

[27] Morera D, Roher N, Ribas L, Balasch JC, Doñate C, Callol A, et al (2011) Rna-seq reveals an integrated immune response in nucleated erythrocytes. PLoS ONE 6. 10.1371/journal.pone.0026998 PMC3203173 22046430

[28] Ciardullo S, Aureli F, Coni E, Guandalini E, Iosi F, Raggi A, et al (2008) Bioaccumulation potential of dietary arsenic, cadmium, lead, mercury, and selenium in organs and tissues of rainbow trout (*Oncorhyncus mykiss*) as a function of fish growth. J Agric Food Chem 56: 2442–2451. 10.1021/jf703572t 18327907

[29] Wood CM. (2011) 1—silver. Fish Physiology 31, Part B: 1–65.

[30] Rayman MP. (2000) The importance of selenium to human health. Lancet 356: 233 10963212 10.1016/S0140-6736(00)02490-9

[31] Hamilton SJ. (2004) Review of selenium toxicity in the aquatic food chain. Sci Total Environ 326: 1–31. 15142762 10.1016/j.scitotenv.2004.01.019

[32] Koller LD, Exon JH. (1986) The two faces of selenium—deficiency and toxicity—are similar in animals and man. Canadian Journal of Veterinary Research 50: 297–306. 3527390 PMC1255217

[33] Hodson PV, Hilton JW. (1983) The nutritional requirements and toxicity to fish of dietary and waterborne selenium. Ecological Bulletins: 335–340.

[34] Gatlin DM, Wilson RP. (1984) Dietary selenium requirement of fingerling channel catfish. J Nutr 114: 627–633. 6699743 10.1093/jn/114.3.627

[35] Hilton JW, Hodson PV, Slinger SJ. (1980) The requirement and toxicity of selenium in rainbow trout (*Salmo gairdneri*). J Nutr 110: 2527–2535. 7441379 10.1093/jn/110.12.2527

[36] Jovanovic A, Grubor-Lajsic G, Djukic N, Gardinovacki G, Matic A, Spasic M. (1997) The effect of selenium on antioxidant system in erythrocytes and liver of the carp (*Cyprinus carpio* L.). Crit Rev Food Sci Nutr 37: 443–448. 9315433 10.1080/10408399709527783

[37] Wang C, Lovell RT. (1997) Organic selenium sources, selenomethionine and selenoyeast, have higher bioavailability than an inorganic selenium source, sodium selenite, in diets for channel catfish (*Ictalurus punctatus*). Aquaculture 152: 223–234.

[38] Zhou X, Wang Y, Gu Q, Li W. (2009) Effects of different dietary selenium sources (selenium nanoparticle and selenomethionine) on growth performance, muscle composition and glutathione peroxidase enzyme activity of crucian carp (*Carassius auratus gibelio*). Aquaculture 291: 78–81.

[39] Küçükbay FZ, Yazlak H, Karaca I, Sahin N, Tuzcu M, Cakmak MN, et al (2009) The effects of dietary organic or inorganic selenium in rainbow trout (*Oncorhynchus mykiss*) under crowding conditions. Aquacult Nutr 15: 569–576.

[40] Sweetman JW, Torrecillas S, Dimitroglou A, Rider S, Davies SJ, Izquierdo MS. (2010) Enhancing the natural defences and barrier protection of aquaculture species. Aquacult Res 41: 345–355.

[41] Schrauzer GN. (2006) Selenium yeast: Composition, quality, analysis, and safety. Pure and Applied Chemistry 78: 105–109.

