# Correction: Selenium Supplementation in Fish: A Combined Chemical and Biomolecular Study to Understand Sel-Plex Assimilation and Impact on Selenoproteome Expression in Rainbow Trout (*Oncorhynchus mykiss*)

**DOI:** 10.1371/journal.pone.0144681

**Published:** 2016-02-10

**Authors:** Davide Pacitti, Muhammad M. Lawan, John Sweetman, Samuel A. M. Martin, Jörg Feldmann, Christopher J. Secombes

The unit used to indicate Selenium concentration appears incorrectly throughout the manuscript. The correct unit is mg Kg^-1^.

The values for Selenium concentrations provided as 0.5, 4, and 8 mg Kg^-1^ throughout the article are incorrect. The correct Selenium concentrations are 0.25, 2, and 4 mg Kg^-1^ respectively.
